# Building Capacity in Institutional Operational Research in Low-Resource Settings: Protocol for an Implementation Research Study

**DOI:** 10.2196/84284

**Published:** 2026-04-13

**Authors:** Sana Ali, G V S Murthy, Kenneth L Bassett, Priya Reddy, Katie Judson, Suzanne Schwartz Gilbert, Abhilash Patra, Anirudh Gaurang Gudlavalleti, Debangana Mahapatra, Hira Pant, Parami Dakhwa, Sarah Jameel, Sarva Priya Pandey, Shailaja Tetali, Sirshendu Chaudhuri, Srinivas Imandi, Suresh Kamalakannan

**Affiliations:** 1 Pragyaan Sustainable Health Outcomes Foundation, Level 2, Kapil Kavuri Hub, Nanakramguda, Financial District, Hyderabad, 500032, India, 91 8978244994; 2 University of British Columbia, Vancouver, BC, Canada; 3 Seva Canada, Vancouver, BC, Canada; 4 Seva Canada, Kathmandu, Nepal; 5 Indian Institute of Public Health Hyderabad, Hyderabad, India; 6 Northumbria University, Newcastle, United Kingdom

**Keywords:** operational research, delivery of health care, evidence-based practice, capacity building, program evaluation

## Abstract

**Background:**

Operational research (OR) in eye care within resource-constrained settings helps develop context-specific solutions to local challenges. Building OR capacity among eye care personnel enables them to independently generate evidence that drives improvements in eye care delivery.

**Objective:**

This protocol describes the extended phase of the Institutional Operational Research Capacity Building (I-ORCB) program designed to train eye care professionals in the fundamentals and applications of OR.

**Methods:**

The I-ORCB program will be conducted in collaboration with Seva Canada and the Pragyaan Sustainable Health Outcomes Foundation over 3 years (2024‐2027). A total of 10 partner eye hospitals in India and Nepal will participate. The training is organized around 7 work packages covering quantitative and qualitative research methods, data management and analysis, community engagement, scientific writing, grant writing, and ethics. The program will be implemented in two sequential phases: (1) protocol development and ethics committee submission and (2) mentoring, data collection, data analysis, and manuscript preparation. Delivery will include workshops, ongoing mentorship, e-resources, and structured monitoring mechanisms. Evaluation will follow the Kirkpatrick model, and cost-effectiveness will be assessed through the change in knowledge among the participants brought about by the workshops.

**Results:**

This program is currently in the implementation phase. As of March 2025, a total of 10 teams have been enrolled in the I-ORCB program. Data collection for program evaluation began in June 2025 and is expected to continue until late 2026. Expected results include enhanced OR competencies among hospital teams, institutional strengthening for research, development of peer-reviewed manuscripts, and improved capacity to apply OR to service delivery challenges. The results of this program are expected to be published in 2027.

**Conclusions:**

The I-ORCB program applies a practical, learning-by-doing approach and is grounded in the Cooke framework for research capacity building. By integrating structured mentorship, institutional ownership, and flexible virtual learning components, the program is expected to strengthen OR culture in low- and middle-income countries and improve the delivery of eye care services in South Asia.

## Introduction

### Background

Health care providers in low- and middle-income countries (LMICs) often face the challenge of delivering care to a large number of patients with limited financial and human resources [1]. Persistent challenges during routine practice—such as overcrowding, lack of awareness among patients about processes and treatment services, supply and data management issues, and poor communication between physicians and patients—affect the quality and efficiency of services, leading to inadequate care, patient attrition, poor treatment adherence, and suboptimal patient outcomes [2]. Operational research (OR) refers to the strategies, interventions, tools, or knowledge that can enhance the quality, coverage, effectiveness, or performance of the health system or programs in which the research is conducted [3]. OR in eye care, especially within resource-limited settings, plays a critical role in identifying targeted solutions to locally relevant challenges [4,5]. This makes OR a valuable approach for decision-makers seeking to enhance eye care services in different contexts, ultimately contributing to improved eye health outcomes [6]. Building capacity in OR is cost-effective in low-resource settings, where it can enable decision-makers to optimize resource allocation and evaluate interventions efficiently [7].

Recognizing this need, the Operational Research Capacity Building (ORCB) program was first launched in 2019 through a collaboration between the Seva Foundation; Seva Canada; and the Indian Institute of Public Health Hyderabad, a subsidiary of the Public Health Foundation of India. This program aimed to build OR capacity in eye care settings in India and Nepal [4]. Since then, 2 phases of the program have been implemented. During the first phase (2019-2021), eye care hospital staff, including clinicians and administrators, in India and Nepal were trained in OR concepts [4]. Building on the success of ORCB-1, the Evidence-Informed Practice program (2023‐2024) introduced the plan-do-study-act framework focusing on health service quality improvement. Both programs promoted a culture of OR and aimed to generate and use evidence for improving eye health services in resource-poor settings.

However, these earlier phases also encountered challenges. ORCB-1 revealed the need for more targeted strategies to address health system–level and patient-centered challenges [8-11]. In ORCB-2, a major limitation was the lack of high-quality data, which could not be resolved during the program period. Participants also expressed the need for more comprehensive training and skilling and diverse learning formats. It was also observed that many of the staff members who were initially trained had left the institutions, and this hampered the institutions’ adoption of OR more widely. A need for institutional strengthening rather than individual strengthening was perceived to be important for the future iterations of the ORCB program so that the institutions could take responsibility for embedding OR principles into their plans.

In response, Seva Canada, a charitable organization whose mission is to restore sight and prevent blindness in LMICs, and Pragyaan Sustainable Health Outcomes (PRASHO) Foundation, a not-for-profit organization that works to develop sustainable health care solutions through public health initiatives grounded in evidence-based practice, launched the Institutional ORCB (I-ORCB) program in October 2024. This phase marks a renewed focus on sustaining an OR environment in LMICs while addressing prior gaps through enhanced training, mentorship, and institutional engagement. A total of 10 partner hospitals—5 from Nepal and 5 from India—were enrolled in the I-ORCB training program, and a preliminary needs assessment was carried out to guide its development.

### Prior Work

A baseline assessment was carried out in February 2025 to March 2025 to understand the OR culture across the 10 partner hospitals. This assessment involved a self-administered questionnaire and an on-site visit by mentors. Key areas of focus included the OR teams’ familiarity with research, the leadership’s interest and commitment to engage in OR activities, and the overall institutional environment to facilitate research. The findings of the needs assessment are summarized below.

All hospital leadership demonstrated a keen interest in building the research skills of their core teams and had a clear understanding of their institutions’ OR priorities. The data records were well maintained in most hospitals. However, a few hospitals were in the initial stages of developing a hospital management information system or faced challenges integrating data across departments.

Although all teams had a foundational understanding of research, it was noted that research concepts such as study designs and developing an analysis plan remained weak. Scientific writing skills were mostly limited to senior faculty members, with core team members expressing a need for upskilling in this area. Structured training and hand-holding were identified as essential to strengthen these skills.

Most teams demonstrated limited proficiency in statistical analysis. While team members were familiar with the basics of Microsoft Excel, they lacked confidence in statistical applications. The mentors noted that time constraints posed a significant challenge, as teams had to allocate time for OR activities amid their clinical responsibilities. Some teams were exploring the option of recruiting dedicated research personnel to support OR efforts.

The needs assessment informed the design of a customized I-ORCB training program and contributed to the development of this protocol by identifying gaps, key challenges, and areas requiring targeted support.

### Objectives

The objectives of the I-ORCB program are as follows:

- To train the team members of each partner hospital in the fundamentals and applications of OR over a 2-year period
- To provide constant mentorship support to each hospital team until the completion of an OR project over a period of 3 years
- To evaluate the cost-effectiveness of the I-ORCB program in the application of OR over a period of 3 years

## Methods

### Program Framework

This skilling program will be conducted in collaboration with Seva Canada and the PRASHO Foundation. It will run for a duration of 3 years, from July 2024 to July 2027. To guide the design and delivery of this program, the research capacity building framework by Cooke [12] will be applied. This framework provides a robust foundation for building sustainable and context-relevant research skills. It was recently adapted to support virtual and hybrid learning environments, incorporating tools such as online conceptual learning sessions, online skill-building workshops, e-mentorship, and e-resource repositories [13]. Such virtual platforms, particularly for mentoring, ease time and distance constraints in multicountry programs [13].

The model by Cooke [12] aligns well with our goal of building OR skills for individuals and organizations. The principles of this model will be specifically aligned with the I-ORCB program as follows:

- OR skills—OR capacity will be built by training the participants in appropriate skills to identify operational challenges and use evidence to generate actionable insights. Mentorship will ensure the application of these skills in real-world settings.
- Context relevant—all OR projects will be rooted in local operational challenges faced by the partner hospitals.
- Multidisciplinary team—each OR team will comprise professionals from both clinical and nonclinical backgrounds, each with their own set of skills and expertise.
- Knowledge sharing—findings from all OR projects will be shared across partner hospitals through online dissemination sessions.
- Self-sustainability—all teams will be supported to grow into future OR leaders and embed OR as a part of routine practice, ensuring a self-sustaining OR culture.
- Enabling environment—the program will help partner hospitals develop an environment conducive for OR, such as support for strengthening data management systems, setting up an ethics committee, and providing guidance in securing grants.
- Equity in training—the program will incorporate equity in its delivery by providing tailored mentorship support to reduce disparities in OR skills between the partner hospitals.
- Ownership—each OR project will be driven by a motivated hospital team with strong support from the institutional leadership. Teams will take full ownership and responsibility for allocating time, mobilizing resources, and leading the project from inception to completion.

### Training Delivery

#### Overview

The training for the I-ORCB program will follow an output-oriented, learning-by-doing approach as recommended by the International Union Against Tuberculosis and Lung Disease and Médecins Sans Frontières, who advocate for building OR capacity over an extended period through structured training in protocol development, data management and analysis, and scientific writing [3,14,15]. In line with this recommendation, the I-ORCB training will be delivered in 2 phases: phase 1 will support protocol development for an OR topic for a study that each hospital will conduct and submission of the protocol to the ethics committee, and phase 2 will facilitate mentoring, data collection, data analysis, manuscript preparation, and submission to peer-reviewed journals.

Following consensus among the collaborating partners and in alignment with the needs of the partner hospitals, 7 work packages have been proposed, integrating key concepts of the ORCB program. The work packages and their expected outcomes are presented in Table 1. These work packages will provide a holistic framework for building OR capacity for eye care in low-resource settings.

The I-ORCB program will be delivered through 4 core components: skilling workshops, mentorship support, e-resources, and monitoring mechanisms.

**Table 1.** Work packages and their expected outcomes.

Work package	Expected outcome
Training in research methods	To provide participants with a comprehensive understanding of types of research, hypothesis generation, and research question formulation; various study designs appropriate to answer the research questions; types of bias; and confounders in research
Training in data management and analysis	To equip participants with the skills necessary for data collection, managing data and conducting primary and secondary data analysis
Training in qualitative research methods	To train participants in conducting qualitative research, including stakeholder interviews and focus group discussions at the population and eye facility levels
Community engagement for research	To orient the participants on the importance of and modalities for community engagement in eye research
Capacity-building workshops on scientific writing	To build participants’ capacity in various aspects of scientific writing
Capacity building for grant writing	To build participants’ capacity in various aspects of grant writing
Orientation on ethics in research	To orient the participants on the importance of the principles and practice of ethics in research

#### Skilling Workshops

The work packages will be delivered in 2 phases over a 3-year period. Each phase will have a series of interactive online skilling workshops conducted over 3 to 4 months. These workshops will be facilitated by experts from Seva Canada, the PRASHO Foundation, and the Indian Institute of Public Health Hyderabad, each bringing expertise from their respective areas.

The workshops will adopt diverse learning methodologies, such as virtual lectures, hands-on activities, and case-based discussions. Each session will last for approximately 2 hours and will be held once or twice a week. Sessions will be scheduled at convenient times to accommodate the busy schedules of the participants and will be adequately spaced to allow teams sufficient time to simultaneously apply the learnings to their particular OR project.

#### e-Mentorship Support

Each team will be assigned a dedicated mentor who will provide consistent guidance throughout both phases of the skilling workshops. While the lectures will cover the fundamental concepts of OR, the application of these learnings will be situation specific and will be supported by the mentors. The mentors will assist with problem-solving and offer hands-on support through regular interactions. The mentors will hold frequent one-on-one sessions with their teams to foster open communication, build trust, and ensure the timely progression of each project milestone.

#### e-Resource Repository

To reinforce learning and enhance accessibility, all training sessions will be recorded, and accompanying materials such as slide decks and reading resources will be uploaded to a digital platform. This repository will be accessible to all participants at any time, allowing them to refer back to key concepts when needed.

#### Monitoring Mechanism

**Figure 1.**
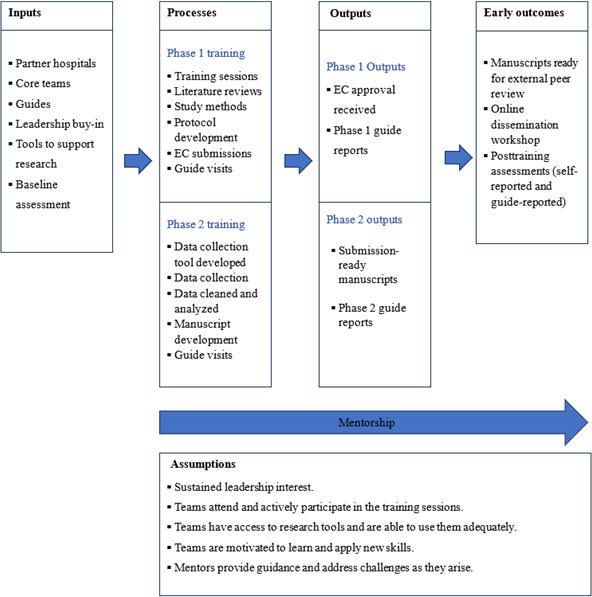
Activity-based logic model. EC: ethics committee.

A monitoring framework has been developed to track the progress toward achieving the program’s objectives. Figure 1 presents the output-oriented logic model for the ORCB program, illustrating how the activities align with the intended outcomes. The corresponding measurement units for each indicator are detailed in Table 2.

**Table 2.** Activity-based indicator matrix.

Measurement	Source
Process indicators: phase 1 training	
Number of research topics developed	Project lead
Number of training sessions conducted	Project in-charge
Number of literature reviews conducted	Hospital feedback form facilitated by the dedicated mentor
Number of study methodologies developed (including research methods, study designs, and ethical considerations)	Hospital feedback form facilitated by the dedicated mentor
Number of data management and analysis plans developed	Hospital feedback form facilitated by the dedicated mentor
Number of protocols developed	Hospital feedback form facilitated by the dedicated mentor
Number of protocols submitted to the EC^a^	Hospital feedback form facilitated by the dedicated mentor
Number of mentor visits completed	Hospital feedback form facilitated by the dedicated mentor
Number of e-mentorship sessions conducted	Project in-charge
Number of e-resources of the training program added to the repository (including PowerPoint presentations and study materials)	Project in-charge
Process indicators: phase 2 training	
Number of data collection tools finalized	Hospital feedback form facilitated by the dedicated mentor
Number of data collection processes completed	Hospital feedback form facilitated by the dedicated mentor
Number of datasets cleaned	Hospital feedback form facilitated by the dedicated mentor
Number of data analyses conducted	Reports submitted to the mentor
Number of mentor visits completed	Project in-charge
Number of manuscripts developed	Submissions to the mentor
Number of manuscripts submitted to journals	Follow-up by the mentor
Number of e-mentorship sessions conducted	Project in-charge
Number of e-resources added to the repository	Project in-charge
Output indicators	
Number of team members trained	Project in-charge
Number of protocols submitted to the EC or number of EC approvals received	Follow-up by the mentor and project in-charge
Number of manuscripts submitted to journals or number of manuscripts developed	Follow-up by the mentor and project in-charge
Phase 1 mentor reports	Phase 1 reports submitted to project in-charge
Phase 2 mentor reports	Phase 2 reports submitted to project in-charge
Early outcome indicators	
Number of publications or number of manuscripts submitted to journals	Project in-charge
Number of projects completed or number of projects disseminated in online workshops	Project in-charge
Posttraining assessment: number of self-reported questionnaires completed and returned	Project in-charge
Posttraining assessment: number of mentor assessments completed	Reports submitted to project in-charge

a EC: ethics committee.

### I-ORCB Evaluation Plan

**Figure 2.**
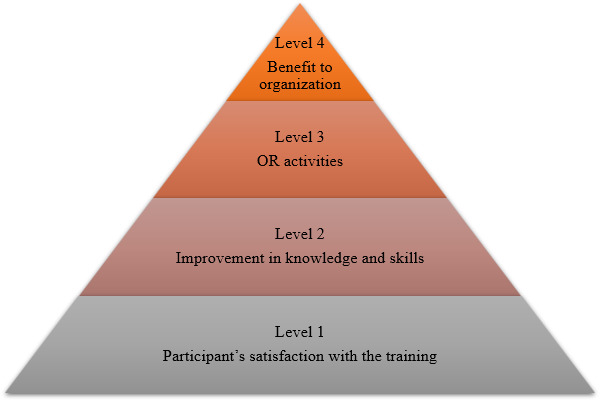
Kirkpatrick model for program evaluation [17]. OR: operational research.

The I-ORCB program will use the Kirkpatrick model for evaluation of training [16]. This model has 4 levels of evaluation, and each level has an impact on the next level (Figure 2 [17]). Level 1 focuses on participants’ satisfaction with the training they received and will be measured through feedback forms. Level 2 focuses on acquiring knowledge and skills and will be measured through pre- and posttraining tests. Level 3 focuses on transferring knowledge and skills to perform OR activities and will be measured through the achievement of activity-based milestones. Level 4 focuses on the main outcome and the benefit to the organization as a result of OR training. This will be measured through completion of an OR project and a final mentor visit.

### Evaluating the Cost-Effectiveness of the I-ORCB Program

The cost-effectiveness of the I-ORCB training program will be evaluated by estimating the cost of delivering the training and the resulting improvement in the OR knowledge of the ORCB teams [18]. The direct costs will include the training facilitators’ fees, training materials, and logistics. The indirect costs will include opportunistic costs (eg, trainee time). The effectiveness of the training will be measured through the change in knowledge among the participants brought about by the workshops.

### Expected Outcomes

On the basis of the gaps identified during the baseline assessment (Table 3), the I-ORCB program aims to achieve the following outcomes: (1) enhanced capacity to use and apply existing data for OR projects, (2) enhanced competencies and skills of the teams in conducting studies using OR approaches to address contextual service-related challenges, (3) partner hospitals becoming the hub for OR and expanding the OR environment by leading and training other individuals in the hospitals or partner eye institutions in OR, and (4) improved eye health services and eye health outcomes brought about through sustained OR capacity building.

**Table 3.** Baseline assessment for operational research (OR; n=10 hospitals).

OR components	Limited capacity, n (%)	Moderate capacity, n (%)	Strong capacity, n (%)
Leadership interest	0 (0)	0 (0)	10 (100)
Research-enabling environment	4 (40)	4 (40)	2 (20)
Data records	2 (20)	6 (60)	2 (20)
Data tools	4 (40)	3 (30)	3 (30)
Statistical proficiency	9 (90)	0 (0)	1 (10)
Data collection skills	3 (30)	1 (10)	6 (60)
Manuscript drafting skills	1 (10)	4 (40)	5 (50)
Basic research skills	0 (0)	5 (50)	5 (50)
Research dissemination skills	0 (0)	1 (10)	9 (90)
Total overall baseline capacity score	0 (0)	9 (90)	1 (10)

### Recruitment

The partner hospitals included in the I-ORCB program were selected based on their prior participation in earlier phases of the ORCB program and interest in continuing their involvement. Additional eye hospitals were also invited, and those who were interested were subsequently enrolled. In total, 10 partner eye hospitals were recruited—5 from India and 5 from Nepal.

Each partner hospital was requested to form a core team comprising a hospital lead to provide leadership support and guide the selection of an OR project; a research lead to directly oversee the planning and implementation of an OR project; and a data lead to oversee data collection, management, and analysis. This core team then recruited supporting members from within the hospital to form an OR team.

### Institutional and Participant Characteristics

The partner eye hospitals represent a mix of urban, semiurban, and rural settings across India and Nepal, serving both local and cross-border populations. These hospitals collectively provide a wide range of ophthalmic services, including cataract services and refractive error correction, as well as advanced subspecialties such as corneal and retinal surgeries, and community outreach activities. The hospitals operate through a network of vision centers, satellite clinics, and secondary hospitals, providing referral linkages and reaching both urban and rural populations.

Each hospital formed a multidisciplinary OR team comprising individuals from both clinical and nonclinical areas, such as ophthalmologists, outpatient department managers, research coordinators, optometrists, IT assistants, quality associates, fellows, pharmacists, cornea specialists, corporate social responsibility heads, and superintendents.

### Data Collection Plan for Program Evaluation

Data will be collected to evaluate the 4 levels of the Kirkpatrick model.

For level 1, a structured postsession feedback form will be developed to assess participants’ satisfaction with the training. This form will use a 5-point Likert scale to rate session content, delivery, discussions, and workshop logistics. Open-ended responses will capture participants’ views on the usefulness of the session, need for additional resources or topics, and further suggestions.

For level 2, a pretest-posttest multiple-choice questionnaire (MCQ) will be designed for each session to measure knowledge gained. The questionnaires will be developed by a session facilitator and will be reviewed by team members to ensure content validity, clarity, and relevance.

Both level 1 and 2 tools will be developed in Google Forms. The MCQ form link will be circulated among the teams at least 1 hour prior to each session. Participants will be requested to provide the completed feedback form and postsession MCQs at the end of each session.

Level 3 will be evaluated through mentor reports that will be provided by the mentors of each team following completion of each training phase. These reports will document the extent to which the participants applied their learnings to OR activities.

Level 4 will be evaluated on completion at the end of phase 2. Each team will submit a final report describing how participation in the I-ORCB program supported the implementation of their OR project and contributed to organizational outcomes.

Data collection for levels 1, 2, and 3 will be conducted consistently across both training phases of the I-ORCB program.

### Data Collection Plan for Evaluation of Program Cost-Effectiveness

The data to assess direct cost will be obtained from institutional records, such as invoices, facilitators’ remuneration, and travel and logistics expenses related to the program. Indirect costs will be estimated based on the time spent by the facilitators in developing training materials and implementing the program (including time spent conducting online workshops and team meetings). Information on time use will be extracted from online session records, meeting logs, and activity schedules. The data to assess effectiveness will be obtained from participant responses to the baseline assessment compared against the end line assessment 6 months after completion of the 2 phases of workshops.

### Data Analysis Plan for Program Evaluation

All quantitative data from levels 1 and 2 will be exported from Google Forms to Microsoft Excel. The dataset will be cleaned and coded prior to transfer into Stata (version 19; StataCorp) for statistical analysis. Descriptive statistics will be used to summarize participant feedback. The Likert scale responses will be reported as frequencies and percentages. For assessing knowledge, the responses will be scored dichotomously (correct=1; incorrect=0).

To compare pre- and posttest scores, a 2-tailed paired or unpaired *t* test will be applied if the data follow a normal distribution. For data not normally distributed, the Wilcoxon signed-rank test or Mann-Whitney *U* test will be used. The results will be reported with 95% CIs and *P* values. A *P* value <.05 will be considered statistically significant. Data will be checked for completeness and accuracy before analysis.

For levels 3 and 4, content analysis will be performed on the reports. The reports will be coded and grouped thematically to reflect learnings and applications of OR.

### Data Analysis Plan for Evaluation of Program Cost-Effectiveness

Program cost-effectiveness will be assessed by calculating the cost-effectiveness ratio, that is, the ratio of total program cost to program effectiveness, using the formula cost-effectiveness ratio = total cost/effectiveness, where cost is the sum of direct and indirect costs and effectiveness is the mean difference between the baseline and end line assessment scores.

### Ethical Considerations

The I-ORCB protocol has received ethics approval from the institutional ethics committee at Pushpagiri Vitreo Retina Institute, Hyderabad, (PVRI/IEC/2025/0009). Consent of participants is implied in the training program. The participants’ right to privacy and confidentiality will be respected. All data will be anonymized prior to analysis and personal identifiers will not be disclosed while reporting the results. The data will be kept secure and will be accessible by authorized personnel only. The participants will receive training in operational research. There is no monetary compensation or financial incentive provided for participating in this training program.

## Results

As of March 2025, a total of 10 teams have been enrolled in the I-ORCB program. This program is currently in the implementation phase, with teams undergoing training and ongoing mentorship. Data collection for program evaluation began in June 2025 and is expected to continue until late 2026. The results of this program are expected to be published in 2027. This study was funded in late 2024. Data analysis has not yet been initiated.

## Discussion

### Expected Findings

The I-ORCB program aims to enhance eye care services in low-resource settings by offering context-specific solutions to eye care providers, as well as to the ancillary health care staff. By equipping the participants with the knowledge and skills required to develop need-based solutions, the program supports evidence-based decision-making to improve eye health care delivery in LMICs. To the best of our knowledge, no other capacity-building initiative has focused exclusively on OR in eye care in the manner presented in this protocol.

In designing this program, we have drawn on a practical, output-based approach endorsed by the International Union Against Tuberculosis and Lung Disease and Médecins Sans Frontières, who have been the pioneers in building OR capacity among health professionals in public health programs in LMICs [3,14,15]. In addition, by adopting the research capacity building principles of the virtual model of the framework by Cooke [12], the I-ORCB program is grounded in proven methodologies and tailored to meet the challenges of delivering OR training across geographically diverse and resource-constrained settings. This design allows for both flexibility and inclusive learning opportunities [12,13].

OR has been widely applied to improve health care delivery across diverse clinical settings, such as dermatology, oncology, physiotherapy, and dialysis services [19-22]. These applications have demonstrated the value of OR in addressing common challenges related to service efficiency and resource use. Comprehensive eye care aims to provide a continuum of services, ranging from health promotion and prevention to treatment and rehabilitation [23]. Most of these services are delivered on an outpatient basis or as daycare procedures, which, in high volume settings, can lead to challenges in service delivery. OR applications have been shown to address these challenges through scheduling and optimizing patient flow; reducing waiting time; and, thus, improving patient satisfaction [24,25].

While earlier iterations of the ORCB program were promising, they also highlighted the need for deeper engagement. The I-ORCB program retains the core mission of building OR capacity in resource-limited settings and introduces new components to address the gaps identified in previous phases. The structured and spaced-out schedule of the I-ORCB workshops will enable participants to gain an in-depth understanding of key OR concepts while accommodating their demanding professional commitments. Long-term mentorship from dedicated professionals supports participants in real-world challenges in OR, and frequent one-on-one interactions maintain program momentum through the completion of an OR project. The e-resource repository gives participants the flexibility to access the training content at their own pace, accommodating different learning speeds and time constraints. By equipping eye personnel with OR skills, the I-ORCB program will contribute to the broader goal of improving eye health outcomes in LMICs.

The limitations of the I-ORCB program are, first, that the program heavily depends on an online mode for upskilling and mentorship. Therefore, the availability of stable internet connectivity is crucial and may affect participation and engagement. Second, although the workshops will be scheduled based on the convenience of the I-ORCB teams, participation may still pose a challenge due to competing responsibilities. Third, the I-ORCB program will be implemented across diverse geographical and institutional settings, each with different levels of administrative support and resource availability. Due to these differences, the outcomes of the program may vary across sites.

### Conclusions

The I-ORCB program offers a structured framework for building OR capacity in low-resource settings. By equipping eye care institutions with the skills needed to generate and use evidence, this program will contribute to the broader goal of improving eye health outcomes in LMICs.

## Abbreviations

I-ORCB: Institutional Operational Research Capacity Building
LMIC: low- and middle-income country
MCQ: multiple-choice questionnaire
OR: operational research
ORCB: Operational Research Capacity Building
PRASHO: Pragyaan Sustainable Health Outcomes
